# Association of fat quality index with head and neck cancer risk: results from a prospective study

**DOI:** 10.3389/fnut.2026.1737854

**Published:** 2026-02-16

**Authors:** Ziyao Zeng, Linghai Zeng, Yi Xiao, Yahui Jiang, Yuxiang Luo, Linglong Peng, Yaxu Wang, Yunhao Tang

**Affiliations:** 1 Department of Gastrointestinal Surgery, The Second Affiliated Hospital of Chongqing Medical University, Chongqing, China; 2 Department of General Surgery, The Third Affiliated Hospital of Chongqing Medical University, Chongqing, China; 3 Centre for Lipid Research and Chongqing Key Laboratory of Metabolism on Lipid and Glucose, The Second Affiliated Hospital of Chongqing Medical University, Chongqing, China; 4 Department of Cardiothoracic Surgery, The First Affiliated Hospital of Chongqing Medical University, Chongqing, China; 5 Department of Cardiology, Cardiovascular Institute, Thoraxcenter, Erasmus University Medical Center, Rotterdam, Netherlands

**Keywords:** cancer prevention, epidemiology, fat quality index, head and neck cancer, prospective cohort study

## Abstract

**Background:**

As head and neck cancer (HNC) incidence rises, prevention demands attention to diet. Yet most studies emphasize total fat intake while overlooking fat quality. To address this gap, we examined the association between fat quality index (FQI) and HNC risk.

**Methods:**

A total of 98,560 participants were included in this study. Hazard ratios (HR) with 95% CIs for overall HNC were estimated using multivariable Cox models. Site-specific analyses were conducted, and fatty-acid components were evaluated. Effect modification was tested across prespecified subgroups. A joint analysis combined FQI tertiles with percent energy from fat. Robustness was assessed through multiple sensitivity analyses.

**Results:**

Over a median 8.8 years, 267 HNC cases occurred. Higher FQI significantly reduced overall HNC risk (fully adjusted HR for Quartile 4 vs. Quartile 1: 0.62; 95% CI, 0.42–0.91; P-trend = 0.011), demonstrating a linear inverse dose–response. Findings were consistent across subgroups and sensitivity analyses. By subsite, associations were strongest for laryngeal cancer (HR for Q4 vs. Q1: 0.43; 95% CI: 0.21–0.88). The joint analysis revealed that higher FQI was associated with lower HNC risk even among individuals with high total fat intake (HR = 0.36; 95% CI: 0.21–0.59), with no significant interaction observed. Higher Monounsaturated fatty acids (MUFA) and polyunsaturated fatty acids (PUFA) intakes were inversely associated with HNC, whereas Saturated fatty acids (SFA) and Trans fatty acids (TFA) were not.

**Conclusion:**

Higher FQI is associated with lower HNC incidence, independent of total fat intake. These findings highlight that prioritizing dietary fat quality over quantity may be a critical strategy for HNC primary prevention.

## 1 Introduction

Head and neck cancer (HNC) is the seventh most common malignancy worldwide, with over 660,000 new cases and approximately 325,000 deaths reported annually (1). Despite ongoing advances in diagnostic and therapeutic strategies, the prognosis for many HNC subtypes remains poor. Alarmingly, the global age-standardized incidence of HNC is projected to continue rising through 2030 (1). In light of these trends and the limited improvement in long-term outcomes, there is increasing emphasis on primary prevention. A substantial proportion of HNC cases are attributable to modifiable lifestyle factors—including tobacco use, alcohol consumption, and diet—highlighting the potential for population-level interventions to reduce disease burden (2, 3).

Among modifiable lifestyle factors, dietary fat intake has garnered sustained attention in the context of cancer prevention, including for HNC (4, 5). Epidemiological evidence has suggested inverse associations between cancer risk and two distinct dietary strategies: one emphasizing high intake of unsaturated fatty acids, as exemplified by the Mediterranean dietary pattern rich in olive oil, nuts, and fatty fish (6, 7); and the other emphasizing restriction of total fat consumption, regardless of fatty acid subtype (8). This apparent convergence of protective effects from conceptually divergent approaches reflects a key ambiguity in the existing literature. Specifically, it remains unclear whether the quantity or the quality of dietary fat plays a more critical role in modulating carcinogenic processes. Most previous studies have primarily focused on total fat intake, often without adequately characterizing the underlying fatty acid composition or distribution (8–10). This methodological limitation underscores the need for refined nutritional metrics that can disentangle the independent effects of fat quality, thereby providing more nuanced insights into the dietary determinants of HNC risk.

To address this gap, recent studies have proposed composite indices to quantify the quality of dietary fat, rather than relying solely on total intake (11, 12). In particular, the fat quality index (FQI) provides a concise, ratio-based measure of dietary fat composition that goes beyond total fat intake by capturing the relative predominance of unsaturated over saturated/trans fatty acids, thereby offering a more integrative exposure metric than single-nutrient indicators (11, 12). Although the FQI has demonstrated practical value in the investigation of chronic conditions such as cardiovascular disease and diabetes, its application in cancer epidemiology—particularly in relation to HNC—remains limited and largely unexplored (12–14).

Against this backdrop, the present study aims to elucidate the association between FQI and risk of HNC through a prospective cohort design. To this end, we leveraged data from the Prostate, Lung, Colorectal, and Ovarian (PLCO) Cancer Screening Trial—a large-scale, population-based study—to investigate this potential relationship with greater precision and epidemiological rigor.

## 2 Materials and methods

### 2.1 Study design

This study utilized data from the Prostate, Lung, Colorectal and Ovarian (PLCO) Cancer Screening Trial, a large-scale randomized controlled trial that enrolled 154,887 participants aged 55–74 years from 10 screening centers across the United States between 1993 and 2001 (15). The PLCO trial was designed to evaluate cancer screening effectiveness in reducing mortality from prostate, lung, colorectal, and ovarian cancers, with participants randomly assigned to either screening intervention or usual care control groups. Follow-up continued through 2009 for cancer incidence and through 2018 for mortality outcomes. As part of the trial protocol, participant information was systematically collected through a Baseline Questionnaire (BQ) and a Dietary History Questionnaire (DHQ).

**Figure 1:**
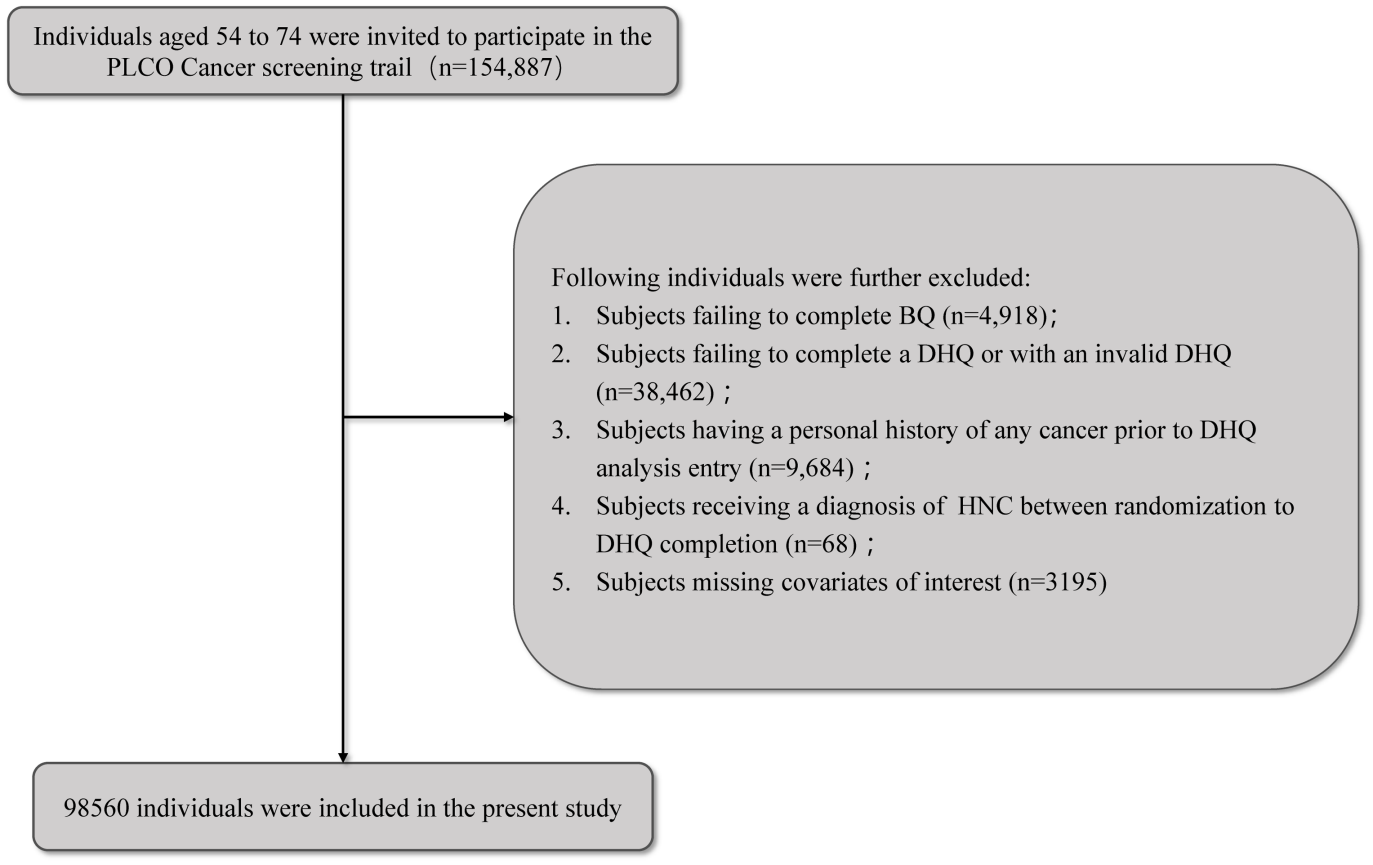
The flow chart of identifying eligible subjects. PLCO, Prostate, Lung, Colorectal, and Ovarian; BQ, baseline questionnaire; DHQ, diet history questionnaire.

To investigate the association between FQI and HNC incidence, we excluded participants with incomplete BQ, missing or invalid DHQ, a history of any cancer before study entry, an HNC diagnosis occurring before completion of the DHQ, or missing key covariates. Participants meeting these criteria were included in the final analytical cohort; the selection process is shown in Figure 1. The original PLCO trial received approval from the National Cancer Institute and Institutional Review Boards at each screening center, with informed consent obtained from all participants. The current analysis was approved by the NCI under Project ID PLCO-1886.

### 2.2 Data collection and FQI score calculation

The participants’ baseline information, including demographic, lifestyle, and clinical factors, was collected through a self-administered BQ. In this study, the following variables were included as covariates: age (continuous), sex (male/female), race (Non-Hispanic White, Non-Hispanic Black, Hispanic, Other), education (some college or less, college graduate, postgraduate), body mass index (BMI; kg/m^2^, calculated as weight in kilograms divided by height in meters squared), pipe smoking status (never, former, current), cigar smoking status (never, former, current), daily cigarette consumption (0 per day, 1–20 per day or >20 per day), alcohol drinking status (no or yes), alcohol consumption (continues, g/days), and family history of HNC (no, yes or possibly).

The DHQ is a validated 137-item self-administered food frequency questionnaire (FFQ) designed to assess dietary intake over the previous 12 months, administered approximately 3 years after enrollment (16). Daily intakes are estimated by combining reported consumption frequency with portion size, while daily energy and nutrient intakes are derived using the nutrient database established from the 1994–1996 US Department of Agriculture Continuing Survey of Individual Intakes (17). The DHQ enables comprehensive evaluation of energy, macronutrient and fatty acid contributions, and fruit and vegetable consumption. Its validity was confirmed in the “Eating at America’s Table” study using 24-h recalls from a nationally representative sample of 1,640 US participants. DHQ performed favorably relative to the other two commonly used FFQs (Block and Willett questionnaires) for absolute intake estimation (16).

The FQI was calculated as the ratio of beneficial fatty acids (monounsaturated fatty acids [MUFA] and polyunsaturated fatty acids [PUFA]) to potentially harmful fatty acids (saturated fatty acids [SFA] and trans fatty acids [TFA]): FQI = (MUFA + PUFA)/(SFA + TFA) (13). Participants were categorized into quartiles based on their FQI scores for analysis.

### 2.3 Ascertainment of HNC

HNC cases were identified through annual study update questionnaires sent to all surviving participants, requesting information on cancer diagnoses including type, date, anatomical site, and healthcare provider details. All self-reported cancers were verified through medical records using standardized data extraction forms. Cancer cases and anatomical locations were confirmed by study physicians blinded to participants’ exposure and risk factor status. HNC was defined according to the International Classification of Diseases for Oncology, Second Edition (ICD-O-2), encompassing oral cavity and pharyngeal cancers (C00.1–C06.9, C09.0–C14.0), laryngeal cancer (C32.0–C32.9), and nasal cavity and middle ear cancers (C30.0, C30.1) (18, 19). The primary outcome was overall HNC incidence. Secondary analyses examined oral cavity/pharyngeal cancer and laryngeal cancer separately.

### 2.4 Statistical analysis

The Cox proportional hazards regression model was used to estimate the hazard ratio (HR) and 95% confidence interval (CI) for the association between FQI and the incidence of HNC, with follow-up duration as the time measure. The follow-up period was defined as the time from completion of the first DHQ to the date of exit, defined as the earliest occurrence of HNC diagnosis, death, loss to follow-up, or end of follow-up (December 31, 2009). To examine the linear trend across FQI quartiles, each participant was assigned the median value of their respective quartile. This value was modeled as a continuous variable in the regression analysis, with the lowest quartile as the reference group. The *p*-value for trend was used to assess the statistical significance of the linear association. Additionally, FQI was modeled as a continuous variable to estimate the risk associated with each unit increase. Based on prior literature and clinical expertise (18, 20, 21), potential confounding factors were identified and included in the Cox regression models to control for confounding. Model 1 adjusted for age, sex, race, and education level. Model 2 further adjusted for family history of HNC, baseline BMI, pipe and cigar smoking status, daily cigarette consumption, alcohol consumption, alcohol use, total protein and carbohydrate intake, energy-adjusted fruit and vegetable intake, and total energy intake.

For secondary outcomes, we applied the same Cox regression framework to assess the associations between FQI and the incidence of site-specific HNC, including oral cavity/pharyngeal cancer and laryngeal cancer. To further explore whether specific types of fatty acids contribute differently to HNC risk, we conducted parallel analyses treating each component of the FQI—monounsaturated, polyunsaturated, saturated, and trans fatty acids—as separate variables in the model. To characterize the dose–response relationship between FQI and HNC incidence across its full range, we fitted restricted cubic spline models with knots placed at the 10th, 50th, and 90th percentiles of FQI distribution (22). The median value of the first quartile (1.27) was used as the reference point. Nonlinearity was assessed by testing the null hypothesis that the coefficient of the second spline term equals zero.

To assess the combined impact of dietary fat quality and quantity on HNC risk, participants were categorized into tertiles based on FQI scores and dichotomized by fat-derived energy percentage (median: 32%), creating six joint exposure groups. Multivariable Cox proportional hazards models estimated hazard ratios (HRs) and 95% confidence intervals (CIs) for HNC incidence, using the lowest FQI tertile with low fat intake (<32%) as the reference. Models were adjusted for covariates described in Model 2. Interaction between FQI and fat intake was tested using a multiplicative term, with significance evaluated by likelihood ratio test.

To explore potential effect modification, we conducted pre-specified subgroup analyses stratified by age (<65 vs. ≥65 years), sex, family history of HNC (absent vs. present/possible), smoking status (pipe: never vs. ever; cigarettes: never vs. ever), daily alcohol consumption (≤1.41 vs. >1.41 g/day), and baseline BMI (<25, 25–30, ≥30 kg/m^2^). Interaction was formally tested by including multiplicative terms in Cox models and comparing nested models using likelihood ratio tests. Within each subgroup, trend tests across FQI quartiles were performed by treating quartile medians as continuous variables in the regression models.

To assess the robustness of our findings, we conducted four sensitivity analyses: (1) excluding participants with implausible energy intake (<500 or >4,000 kcal/day) to minimize dietary reporting errors; (2) excluding those with extreme BMI values (top and bottom 1%) to reduce potential confounding by severe underweight or obesity; (3) excluding HNC cases diagnosed within the first 2 years of follow-up to minimize potential reverse causation; and (4) excluding participants with family history of HNC to focus on sporadic cases. Statistical analyses were performed using R software (version 4.4.2) with survival and splines packages. All tests were two-sided with statistical significance set at *p* < 0.05.

## 3 Results

### 3.1 Baseline characteristics of participants

After applying the exclusion criteria, a final cohort of 98,560 participants was included in the analysis (Figure 1). The mean age of the participants was 65.5 (Standard Deviation, 5.7) years, and they were followed for a mean duration of 8.8 (SD, 1.9) years. The baseline characteristics of the study population, stratified by quartiles of the FQI, are detailed in Table 1. Significant differences were observed across FQI quartiles. Specifically, individuals in the highest FQI quartile (Q4), compared to those in the lowest (Q1), were more likely to be female, younger, and have a college degree or higher. They also reported less family history of HNC and a lower prevalence of heavy smoking. As would be expected by its definition, a higher FQI score was directly correlated with higher intakes of MUFAs and PUFAs, and inversely with SFAs and TFAs. Beyond these constitutive associations, individuals with a higher FQI also consumed more vegetables and fruits, and had a lower total energy intake.

**Table 1:** Baseline characteristics of study population according to overall fat quality index.

Characteristics	Overall	Quartiles of overall fat quality index			
		Quartile 1 (<1.4)	Quartile 2 (1.4–1.6)	Quartile 3 (1.6–1.8)	Quartile 4 (>1.8)
Number of participants	98,560	24,640	24,640	24,641	24,639
*Demographic factors*					
Age	65.5 ± 5.7	65.5 ± 5.8	65.3 ± 5.7	65.5 ± 5.7	65.7 ± 5.7
Male	47,983 (48.7%)	13,373 (54.3%)	12,684 (51.5%)	11,555 (46.9%)	10,371 (42.1%)
Racial/ethnic group					
Non-Hispanic White	89,725 (91.0%)	23,431 (95.1%)	23,252 (94.4%)	22,711 (92.2%)	20,331 (82.5%)
Non-Hispanic Black	3,204 (3.3%)	579 (2.3%)	645 (2.6%)	844 (3.4%)	1,136 (4.6%)
Hispanic	1,427 (1.4%)	364 (1.5%)	358 (1.5%)	350 (1.4%)	355 (1.4%)
Other race/ethnicity ^1^	4,204 (4.3%)	266 (1.1%)	385 (1.6%)	736 (3.0%)	2,817 (11.4%)
Education level					
Some college or less	62,756 (63.7%)	17,036 (69.1%)	16,068 (65.2%)	15,363 (62.3%)	14,289 (58.0%)
College graduate	17,359 (17.6%)	3,884 (15.8%)	4,212 (17.1%)	4,469 (18.1%)	4,794 (19.5%)
Postgraduate	18,445 (18.7%)	3,720 (15.1%)	4,360 (17.7%)	4,809 (19.5%)	5,556 (22.5%)
*Lifestyle and clinical factors*					
Body mass index at baseline (kg/m^2^)	27.2 ± 4.8	27.6 ± 4.9	27.6 ± 4.8	27.3 ± 4.8	26.4 ± 4.7
Pipe smoking status					
Never	84,704 (85.9%)	20,945 (85.0%)	20,937 (85.0%)	21,191 (86.0%)	21,631 (87.8%)
Current	899 (0.9%)	294 (1.2%)	252 (1.0%)	199 (0.8%)	154 (0.6%)
Former	12,957 (13.1%)	3,401 (13.8%)	3,451 (14.0%)	3,251 (13.2%)	2,854 (11.6%)
Cigar smoking status					
Never	86,433 (87.7%)	21,310 (86.5%)	21,374 (86.7%)	21,688 (88.0%)	22,061 (89.5%)
Current	1,637 (1.7%)	508 (2.1%)	444 (1.8%)	357 (1.4%)	328 (1.3%)
Former	10,490 (10.6%)	2,822 (11.5%)	2,822 (11.5%)	2,596 (10.5%)	2,250 (9.1%)
Daily cigarette consumption					
0	46,919 (47.6%)	11,399 (46.3%)	11,655 (47.3%)	11,744 (47.7%)	12,121 (49.2%)
1–20	32,305 (32.8%)	7,830 (31.8%)	7,969 (32.3%)	8,116 (32.9%)	8,390 (34.1%)
>20	19,336 (19.6%)	5,411 (22.0%)	5,016 (20.4%)	4,781 (19.4%)	4,128 (16.8%)
Drink alcohol					
No	26,944 (27.3%)	7,222 (29.3%)	6,460 (26.2%)	6,357 (25.8%)	6,905 (28.0%)
Yes	71,616 (72.7%)	17,418 (70.7%)	18,180 (73.8%)	18,284 (74.2%)	17,734 (72.0%)
Alcohol consumption (g/d)	9.6 ± 25.3	9.4 ± 26.5	9.7 ± 26.5	9.8 ± 24.6	9.4 ± 23.7
Family history of HNC					
No	94,584 (96.0%)	23,492 (95.3%)	23,631 (95.9%)	23,679 (96.1%)	23,782 (96.5%)
Yes	1,413 (1.4%)	368 (1.5%)	358 (1.5%)	355 (1.4%)	332 (1.3%)
Possibly	2,563 (2.6%)	780 (3.2%)	651 (2.6%)	607 (2.5%)	525 (2.1%)
*Dietary factors*					
Total energy intake (kcal/d)	1739.1 ± 736.1	1814.4 ± 786.8	1776.5 ± 755.0	1718.1 ± 714.6	1647.6 ± 672.1
Carbohydrate, %E	52.0 ± 9.4	51.1 ± 9.0	51.9 ± 9.1	52.4 ± 9.4	52.5 ± 10.2
Protein, %E	15.4 ± 3.0	15.3 ± 3.0	15.6 ± 2.9	15.6 ± 2.9	15.1 ± 3.0
Total fat, % E	31.7 ± 7.6	32.7 ± 7.2	31.5 ± 7.3	31.0 ± 7.5	31.8 ± 8.3
Monounsaturated fat acids, %E	11.9 ± 3.2	11.9 ± 2.8	11.9 ± 3.0	11.7 ± 3.1	12.2 ± 3.7
Polyunsaturated fat acids, %E	7.2 ± 2.2	5.9 ± 1.5	6.8 ± 1.6	7.4 ± 1.9	8.8 ± 2.6
Saturated fat acids, %E	10.1 ± 3.0	12.3 ± 3.0	10.3 ± 2.5	9.3 ± 2.4	8.3 ± 2.3
Trans-fatty acids, %E	2.0 ± 0.7	2.2 ± 0.7	2.2 ± 0.7	2.1 ± 0.7	1.7 ± 0.7
Vegetables intake (g/day)	284.0 ± 186.0	244.6 ± 162.1	277.0 ± 172.3	296.1 ± 184.3	318.3 ± 213.3
Fruits intake (g/day)	177.9 ± 150.3	151.6 ± 134.1	170.5 ± 141.0	183.5 ± 148.9	206.1 ± 169.5

%E, the percentage of total energy intake. HNC, head and neck cancer. Values are shown as mean (standard deviation) unless stated otherwise. 1: “Other race/ethnicity” refers to Asian, Pacific Islander, or American Indian.

### 3.2 Association between HNC incidence and FQI score

**Figure 2:**
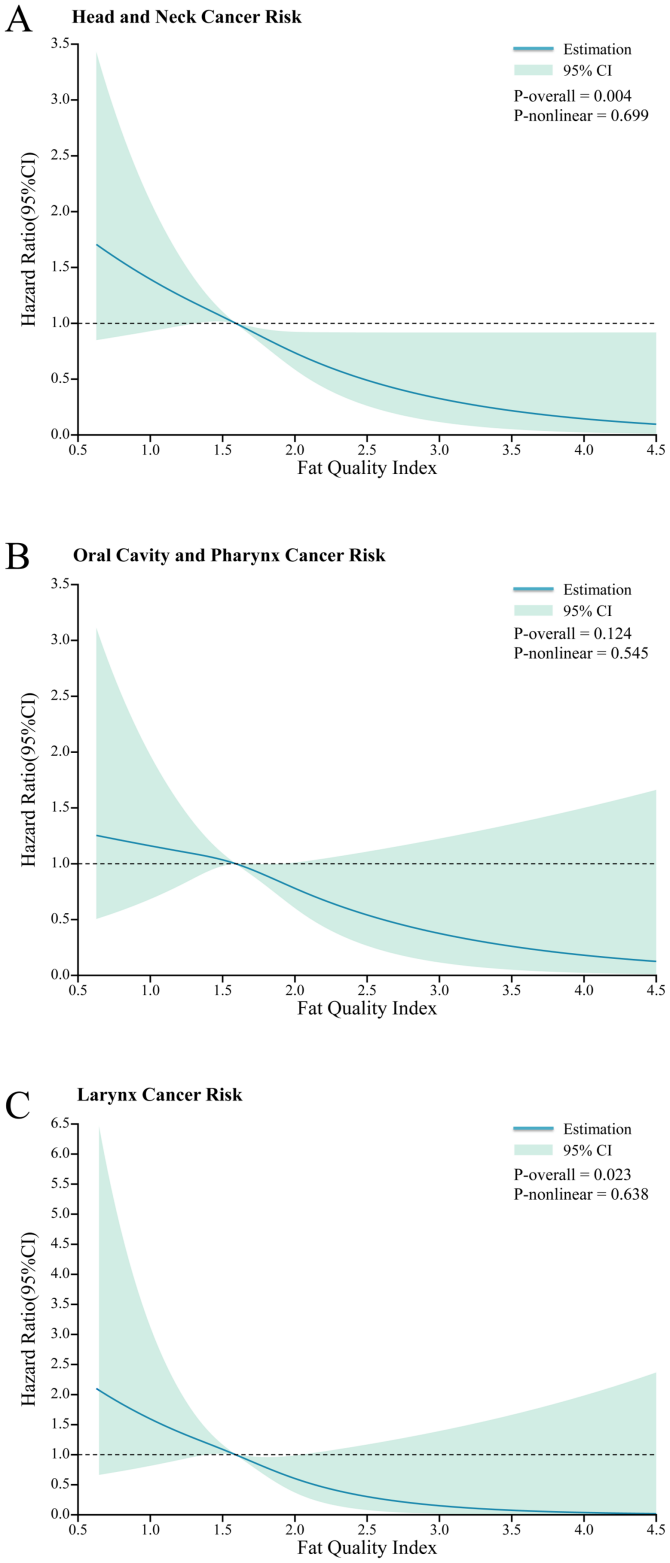
Nonlinear dose–response analysis on the association of FQI score with the risk of HNC (**A**: all HNC; **B**: oral cavity and pharynx cancer; **C**: larynx cancer), hazard ratio was adjusted for age, sex, race, education level and family history of HNC, baseline BMI, pipe and cigar smoking status, daily cigarette consumption, alcohol consumption, alcohol use, total protein and carbohydrate intake, energy-adjusted fruit and vegetable intake, and total energy intake.

Over a mean follow-up of 8.8 ± 1.9 years, corresponding to 872,167 person-years, a total of 267 incident cases of HNC were documented, including 166 cases of oral cavity and pharyngeal cancer, 91 cases of laryngeal cancer, and 10 cases involving other anatomical subsites. This yields an overall incidence rate of approximately 0.306 per 1,000 person-years. In the primary analysis treating FQI as a categorical variable, a significantly lower HNC risk was observed in the highest FQI quartile compared to the lowest (fully adjusted HR for Q4 vs. Q1: 0.62; 95% CI, 0.42–0.91), with a significant dose–response trend across quartiles (P-trend = 0.011). This inverse association was consistent when FQI was modeled as a continuous variable, where each standard deviation increase in the FQI score was associated with a 22% lower risk of HNC (fully adjusted HR: 0.78; 95% CI, 0.68–0.90). To further characterize this relationship, a restricted cubic spline analysis revealed a linear inverse dose–response relationship between FQI and HNC risk, with no evidence of nonlinearity (P-nonlinearity = 0.699; Figure 2A) per SD (Table 2).

**Table 2:** Hazard ratios and 95% confidence interval of the association between FQI and head and neck cancer incidence.

Outcome	FQI, Hazard ratios (95% confidence interval)				*P* for trend ^1^	Continuous (per SD increment)
	Quartile 1 (lowest)	Quartile 2	Quartile 3	Quartile 4 (highest)		
FQI, median (IQR)	1.27 (1.17–1.34)	1.50 (1.45–1.54)	1.69 (1.64–1.75)	2.02 (1.90–2.24)		
Person-years	215,918	218,294	219,166	218,789		
Head and Neck^2^						
Cases, *n*	80	81	64	42		
Incidence rate (95% CI)^3^	0.37 (0.30, 0.46)	0.37 (0.30, 0.46)	0.29 (0.23, 0.37)	0.19 (0.14, 0.26)		
Unadjusted	1.00 (reference)	1.00 (0.73, 1.36)	0.79 (0.57, 1.09)	0.52 (0.36, 0.75)	<0.001	0.73 (0.63, 0.84)
Model 1^3^	1.00 (reference)	1.25 (0.92, 1.68)	0.86 (0.61, 1.20)	0.53 (0.36, 0.79)	0.002	0.79 (0.68, 0.91)
Model 2 ^4^	1.00 (reference)	1.10 (0.80, 1.49)	0.91 (0.65, 1.27)	0.62 (0.42, 0.91)	0.011	0.78 (0.68, 0.90)
Oral cavity and Pharynx						
Cases, *n*	46	43	46	31		
Incidence rate (95% CI) ^4^	0.21 (0.16, 0.28)	0.20 (0.15, 0.27)	0.21 (0.16, 0.28)	0.14 (0.10, 0.20)		
Unadjusted	1.00 (reference)	0.92 (0.61, 1.40)	0.99 (0.65, 1.48)	0.67 (0.42, 1.05)	0.099	0.80 (0.67, 0.95)
Model 1 ^3^	1.00 (reference)	0.96 (0.63, 1.46)	1.07 (0.71, 1.62)	0.76 (0.48, 1.22)	0.334	0.84 (0.70, 1.00)
Model 2 ^4^	1.00 (reference)	1.00 (0.66, 1.52)	1.12 (0.74, 1.69)	0.75 (0.47, 1.20)	0.304	0.83 (0.70, 0.99)
Larynx						
Cases, *n*	31	35	15	10		
Incidence rate (95% CI)^5^	0.14 (0.10, 0.20)	0.16 (0.12, 0.22)	0.07 (0.04, 0.11)	0.05 (0.02, 0.08)		
Unadjusted	1.00 (reference)	1.12 (0.69, 1.81)	0.48 (0.26, 0.88)	0.32 (0.16, 0.65)	<0.001	0.61 (0.47, 0.78)
Model 1^3^	1.00 (reference)	1.19 (0.73, 1.93)	0.56 (0.30, 1.04)	0.44 (0.21, 0.91)	0.007	0.69 (0.53, 0.89)
Model 2 ^4^	1.00 (reference)	1.26 (0.77, 2.04)	0.58 (0.31, 1.08)	0.43 (0.21, 0.88)	0.005	0.69 (0.53, 0.89)

FQI, fat quality index; SD, standard deviation. ^1^Trend test was performed using median value of FQI quintile as a continuous variable. ^2^Including 166 oral cavity and pharynx cancer cases, 91 larynx cancer cases and 10 nasal cavity and middle ear cancer cases. ^3^Incidence rate was calculated per 1,000 person-years. ^4^Model 1 was controlled with age (continuous), sex (male, female), race (Non-Hispanic White, Non-Hispanic Black, Hispanic, other race/ethnicity), education levels (some college or less, college graduate, postgraduate). ^5^Model 2 was additionally controlled with family history of head and neck cancer (no, yes, possibly), body mass index at baseline (continuous), pipe smoking status (never, current or former), cigar smoking status (never, current or former), daily cigarette consumption (0, 1–20, >20), alcohol consumption (continuous), drink alcohol (no, yes), total protein intake (continuous), total carbohydrates intake (continuous), energy-adjusted consumption of vegetables (continuous), energy-adjusted consumption of fruits (continuous) and total energy intake (continuous).

When examining HNC by anatomical subsite, the inverse association was most pronounced for laryngeal cancer. In fully adjusted models, both categorical (HR for Q4 vs. Q1: 0.43; 95% CI: 0.21–0.88; P-trend = 0.05) and continuous analyses (HR per SD: 0.69; 95% CI: 0.53–0.89) revealed a significant protective effect of higher FQI. For oral cavity and pharyngeal cancer, a similar inverse trend was observed, although the association was not statistically significant when FQI was treated as a categorical variable (HR for Q4 vs. Q1: 0.75; 95% CI, 0.47–1.20). However, the continuous analysis did yield a significant association (HR per SD: 0.83; 95% CI, 0.70–0.99). Despite some differences in statistical significance, restricted cubic spline models indicated linear inverse dose–response relationships for both subsites (P-nonlinearity > 0.05 for both; Figures 2B,C).

**Figure 3:**
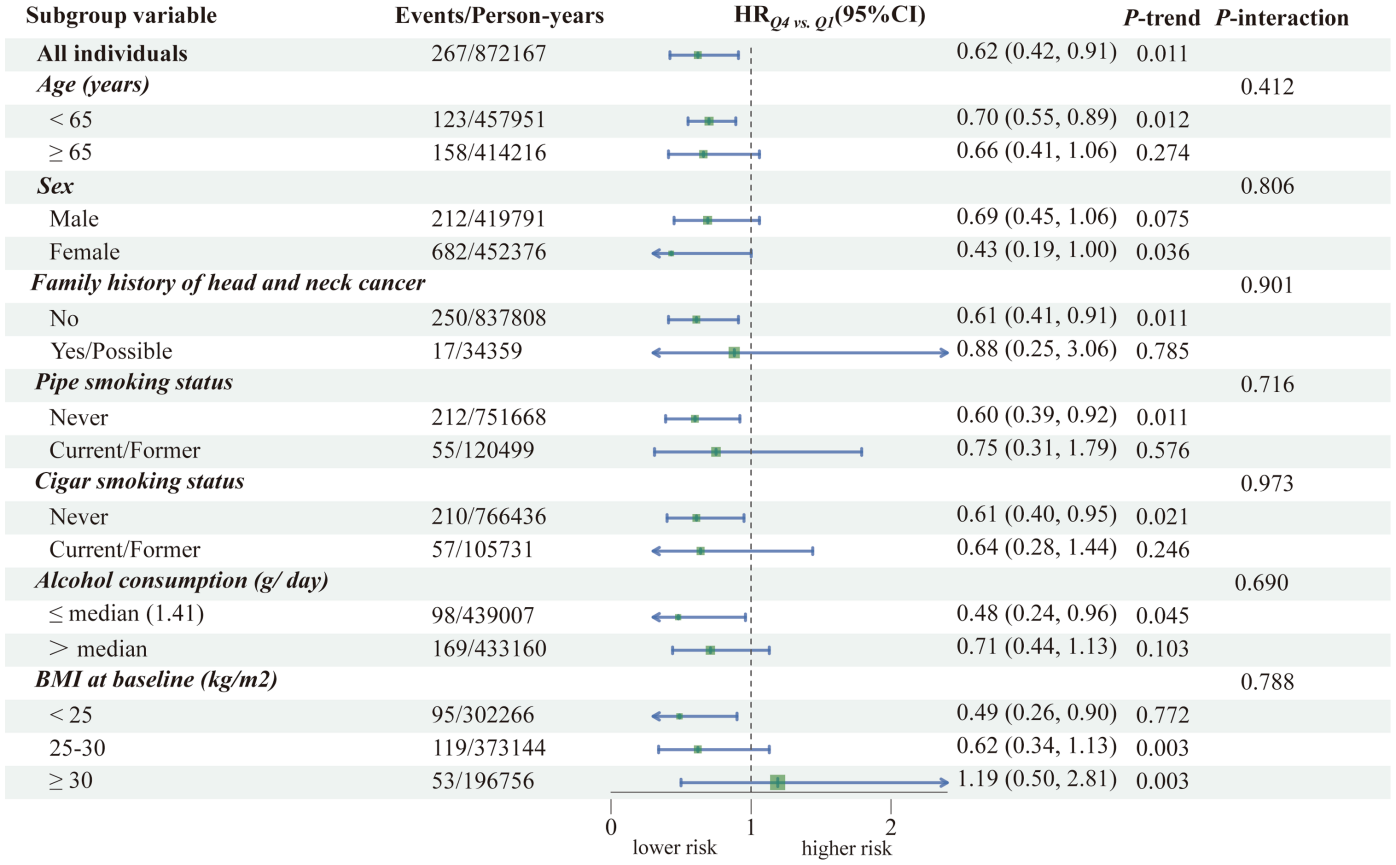
Subgroup analysis and Cox proportional hazards model. A forest plot was generated to assess subgroup effects stratified by age, sex, family history of HNC, smoking status, daily alcohol consumption, and baseline BMI. A Cox proportional hazards model was employed, adjusting for age, sex, race, education level, family history of HNC, baseline BMI, pipe and cigar smoking status, daily cigarette consumption, alcohol consumption, total protein and carbohydrate intake, energy-adjusted fruit and vegetable intake, and total energy intake. Interaction terms were incorporated into the model, and likelihood ratio tests were performed to compare nested models for interaction testing. Trend tests across quartiles of the FQI were conducted by including the median of each quartile as a continuous variable in the regression model.

In further subgroup analyses, the inverse association between FQI and HNC risk remained consistent across various participant characteristics, including age, sex, family history of HNC, smoking status, alcohol consumption, and baseline BMI. We found no evidence of statistically significant effect modification by any of these factors (all *p*-values for interaction > 0.05; Figure 3), suggesting the robustness of our primary finding.

### 3.3 Additional analysis

The associations between different subtypes of dietary fatty acids and the risk of HNC and its anatomical subsites are presented in Table 3. A higher intake of MUFA was significantly associated with a reduced risk of HNC, with individuals in the highest quartile of MUFA consumption exhibiting a 45% lower risk compared to those in the lowest quartile (HR = 0.55; 95% CI: 0.33–0.91; *p* for trend = 0.012). A similar inverse association was also evident when MUFA intake was treated as a continuous variable (HR per sd increment = 0.75; 95% CI: 0.61–0.92). These protective effects extended to cancers of the oral cavity and pharynx (HR for Quartile 4 vs. Quartile 1 = 0.45; 95% CI: 0.24–0.84; *p* for trend = 0.016; HR per SD increment = 0.72; 95% CI: 0.56–0.93), while no statistically significant association was found for laryngeal cancer. A parallel pattern was observed for PUFA, with the highest PUFA intake quartile associated with a substantially decreased risk of HNC (HR for Quartile 4 vs. Quartile 1 = 0.63; 95% CI: 0.41–0.95; p for trend = 0.013; HR per SD increment = 0.77; 95% CI: 0.66–0.91). This protective association extended to oral cavity and pharyngeal cancer when PUFA was treated as a continuous variable (HR per SD increment = 0.76; 95% CI: 0.62–0.92), while the comparison of extreme quartiles showed a similar but non-significant effect (HR = 0.61; 95% CI: 0.36–1.05; p for trend = 0.027). In contrast, no significant associations were detected between SFA and HNC or its subsites. Similarly, TFA showed no meaningful correlation with HNC incidence, although a non-significant elevated risk was noted among participants in the highest TFA quartile (HR for Quartile 4 vs. Quartile 1 = 1.30; 95% CI: 0.96–1.76; p for trend = 0.359).

**Table 3:** Dietary fatty acids intake and the risk of head and neck cancer or subsites in the PLCO cohort.

Types of dietary fatty acids	Head and neck		Oral cavity and Pharynx		Larynx	
	Cohort/Cases	HR (95% CI) ^1^	Cohort/Cases	HR (95% CI) ^1^	Cohort/Cases	HR (95% CI) ^1^
Total MUFAs (%E)						
Q1 (<9.8)	24,640/61	1.00 (reference)	24,640/45	1.00 (reference)	24,640/16	1.00 (reference)
Q2 (9.8–11.9)	24,640/67	0.94 (0.64, 1.37)	24,640/36	0.70 (0.43, 1.12)	24,640/20	1.35 (0.69, 2.64)
Q3 (11.9–14.0)	24,640/66	0.72 (0.47, 1.09)	24,640/43	0.66 (0.39, 1.09)	24,640/27	0.72 (0.33, 1.57)
Q4 (>14.0)	24,640/73	0.55 (0.33, 0.91)	24,640/42	0.45 (0.24, 0.84)	24,640/28	0.65 (0.26, 1.65)
*P* for trend ^2^		**0.012**		**0.016**		0.169
Per SD increment		0.75 (0.61, 0.92)		0.72 (0.56, 0.93)		0.79 (0.55, 1.15)
Total PUFAs (%E)						
Q1 (<5.76)	24,640/76	1.00 (reference)	24,640/46	1.00 (reference)	24,640/27	1.00 (reference)
Q2 (5.76–7.00)	24,640/74	0.99 (0.70, 1.38)	24,640/50	1.15 (0.75, 1.76)	24,640/22	0.77 (0.43, 1.40)
Q3 (7.00–8.45)	24,640/62	0.77 (0.54, 1.12)	24,640/37	0.79 (0.49, 1.27)	24,640/23	0.76 (0.41, 1.41)
Q4 (>8.45)	24,640/55	0.63 (0.41, 0.95)	24,640/33	0.61 (0.36, 1.05)	24,640/19	0.60 (0.30, 1.21)
*P* for trend		**0.013**		**0.027**		0.172
Per SD increment		0.77 (0.66, 0.91)		0.76 (0.62, 0.92)		0.80 (0.61, 1.05)
Total SFAs (%E)						
Q1 (<7.95)	24,640/56	1.00 (reference)	24,640/40	1.00 (reference)	24,640/15	1.00 (reference)
Q2 (7.95–9.89)	24,640/60	1.04 (0.70, 1.55)	24,640/33	0.81 (0.49, 1.34)	24,640/24	1.50 (0.74, 3.03)
Q3 (9.89–11.95)	24,640/72	1.09 (0.72, 1.66)	24,640/51	1.10 (0.67, 1.83)	24,640/17	0.92 (0.41, 2.06)
Q4 (>11.95)	24,640/79	0.95 (0.59, 1.54)	24,640/42	0.73 (0.40, 1.33)	24,640/35	1.49 (0.64, 3.46)
*P* for trend		0.789		0.426		0.528
Per SD increment		1.08 (0.90, 1.28)		0.96 (0.77, 1.20)		1.04 (0.92, 1.17)
Total TFAs (%E)						
Q1 (<1.54)	24,640/53	1.00 (reference)	24,640/35	1.00 (reference)	24,640/17	1.00 (reference)
Q2 (1.54–1.96)	24,640/67	1.31 (0.89, 1.93)	24,640/43	1.38 (0.85, 2.23)	24,640/19	1.03 (0.52, 2.05)
Q3 (1.96–2.43)	24,640/66	1.19 (0.80, 1.78)	24,640/39	1.18 (0.71, 1.97)	24,640/26	1.25 (0.64, 2.46)
Q4 (>2.43)	24,640/81	1.30 (0.86, 1.96)	24,640/49	1.34 (0.79, 2.25)	24,640/29	1.19 (0.59, 2.40)
*P* for trend		0.359		0.438		0.582
Per SD increment		1.07 (0.93, 1.23)		1.08 (0.90, 1.28)		1.05 (0.83, 1.33)

SD, standard deviation; %E, the percentage of total energy intake; HR, hazard ratios; CI, confidence interval. MUFAs, monounsaturated fatty acids; PUFAs, polyunsaturated fatty acids; SFAs, saturated fatty acids; TFAs, trans-fatty acids. The SD value of MUFAs, PUFAs, SFAs, and TFAs is 3.16, 2.19, 3.00, and 0.69, separately. ^1^HR was adjusted for age (continuous), sex (male, female), race (Non-Hispanic White, Non-Hispanic Black, Hispanic, other race/ethnicity), education levels (some college or less, college graduate, postgraduate), family history of head and neck cancer (no, yes, possibly), body mass index at baseline (continuous), pipe smoking status (never, current or former), cigar smoking status (never, current or former), daily cigarette consumption (0, 1–20, >20), alcohol consumption (continuous), drink alcohol (no, yes), total protein intake (continuous), total carbohydrates intake (continuous), energy-adjusted consumption of vegetables (continuous), energy-adjusted consumption of fruits (continuous) and total energy intake (continuous). ^2^Trend test was performed using median value of dietary fatty acids quintile as a continuous variable. Bold values indicate statistically significant results (*p* < 0.05).

**Figure 4:**
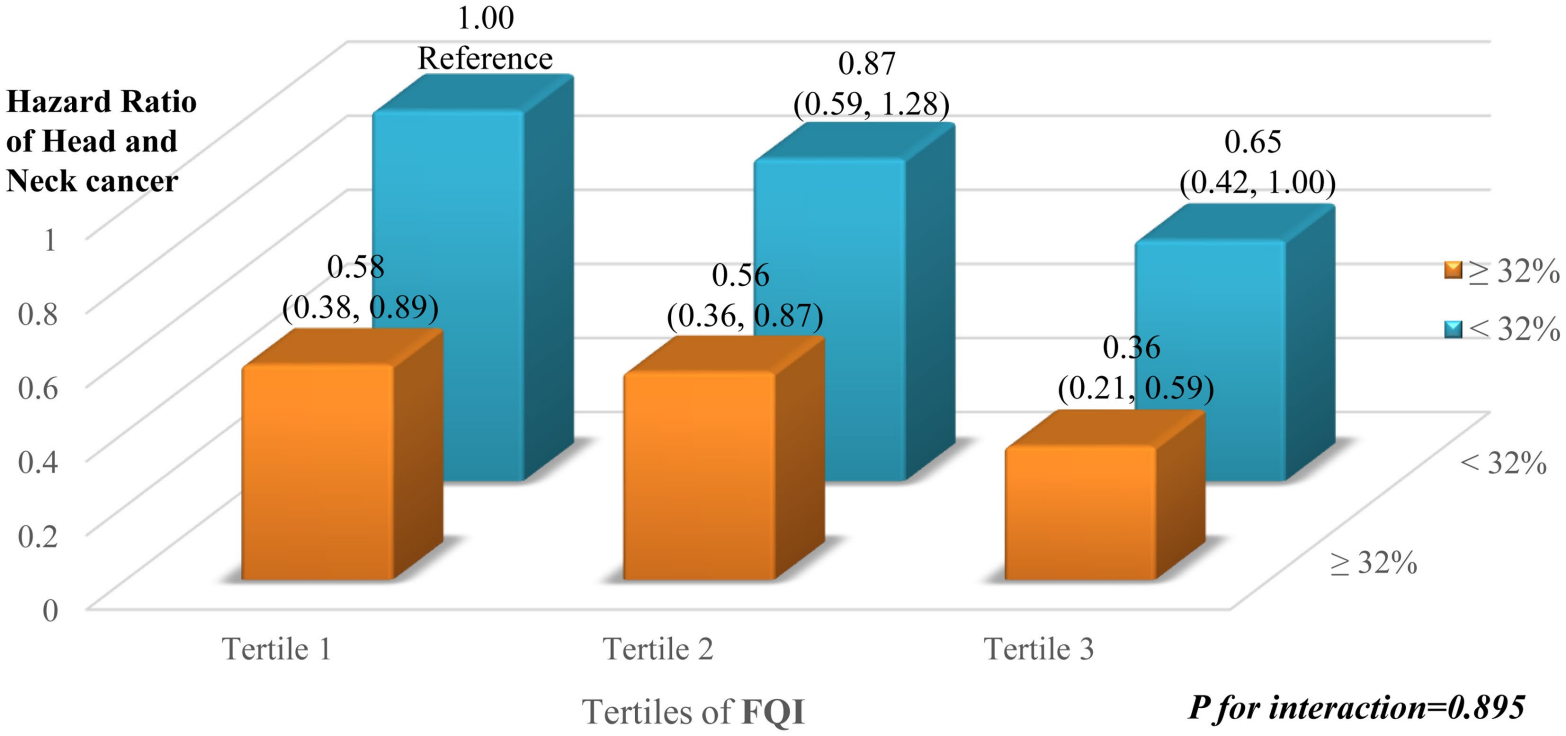
The six combined exposure groups were formed based on the joint classification of FQI score and fat-derived energy percentage (median: 32%), with the lowest fat mass index group and low fat intake (<32%) serving as the reference. The HRs and 95% CIs for HNC risk were calculated. The model was adjusted for age, sex, race, education level, family history of HNC, baseline BMI, pipe and cigar smoking status, daily cigarette consumption, alcohol consumption, total protein and carbohydrate intake, energy-adjusted fruit and vegetable intake, and total energy intake.

Figure 4 illustrates the HR for HNC according to the joint stratification by the tertiles of the FQI and the proportion of energy intake from fat (<32% vs. ≥32%). Across all FQI tertiles, individuals with a higher proportion of energy from fat (≥32%) demonstrated consistently lower HR of HNC compared to their counterparts with lower fat intake. Notably, in the highest FQI tertile (Tertile 3), the HR for HNC was substantially lower among participants with fat intake ≥32% (HR = 0.36; 95% CI: 0.21–0.59), relative to the reference group (Tertile 1 with fat <32%). However, no significant interaction was observed between dietary fat intake and FQI on HNC risk (p for interaction = 0.895), suggesting that the protective association of higher FQI is not strongly modified by the level of fat consumption.

### 3.4 Sensitivity analyses

A series of sensitivity analyses were conducted to evaluate the robustness of the primary findings. Overall, the inverse association between FQI and the risk of HNC remained largely unchanged across various analytical scenarios.

## 4 Discussion

In this prospective analysis based on the PLCO Cancer Screening Trial, we investigated the association between FQI and the risk of HNC. Higher FQI scores—reflecting a greater intake of unsaturated fats relative to saturated and trans fats—were significantly associated with a reduced overall risk of HNC. This inverse association was particularly pronounced for laryngeal cancer, whereas no significant relationship was observed for cancers of the oral cavity and pharynx. Restricted cubic spline models further demonstrated a linear dose–response relationship between increasing FQI and decreasing incidence of both HNC and its laryngeal subtype. Importantly, the observed protective association was consistent across key subgroups, including by smoking status and alcohol consumption, and remained robust in a series of sensitivity analyses.

Based on a review of the available literature, very few studies have investigated the FQI in the context of cancer. One notable example is a prospective study by Zheng et al. on 701 ovarian cancer patients, which reported that a higher FQI was associated with lower all-cause mortality (23). In the field of HNC, research has traditionally focused on fat *quantity* rather than *quality*. For instance, an analysis of the PLCO cohort by Wang et al. suggested that adherence to a low-fat diet was associated with a reduced risk of HNC, particularly laryngeal cancer (8). However, by focusing solely on total fat intake, that approach does not disentangle the distinct health effects of different fat types, such as saturated versus unsaturated fats (24–26). While the Mediterranean diet, which is characterized by a high FQI, has been linked to a lower HNC risk in previous studies (27, 28), it encompasses a wide range of other nutritional factors beyond fat quality (e.g., high intake of fruits, vegetables, and whole grains) (6, 29). Therefore, a clear gap existed in the literature regarding the specific role of dietary fat *quality*, as measured directly by the FQI, on HNC incidence. Our study is the first large-scale prospective investigation to address this gap, and to our knowledge, the first to report such an association in a cohort of over 90,000 participants (Table 4).

**Table 4:** Sensitivity analyses on the between fat quality index and head and neck cancer incidence.

Categories	Cohort/Cases	HR _Quartile 4 vs. Quartile 1_ (95% CI) ^1^	*P* for trend ^2^
Primary analysis	98,560/267	0.62 (0.42, 0.91)	0.011
Excluding participants with extreme energy intake ^3^	97,123/259	0.66 (0.44, 0.97)	0.022
Excluding participants with extreme BMI (baseline) ^4^	96,619/262	0.64 (0.43, 0.94)	0.017
Excluded cases observed within the first 1 years of follow-up	98,542/249	0.69 (0.46, 1.02)	0.030
Excluded cases observed within the first 2 years of follow-up	98,509/216	0.65 (0.43, 0.99)	0.026
Excluded participants with family history of head and neck cancer	94,584/250	0.61 (0.41, 0.91)	0.011

^1^HR was adjusted for age (continuous), sex (male, female), race (Non-Hispanic White, Non-Hispanic Black, Hispanic, other race/ethnicity), education levels (some college or less, college graduate, postgraduate), family history of head and neck cancer (no, yes, possibly), body mass index at baseline (continuous), pipe smoking status (never, current or former), cigar smoking status (never, current or former), daily cigarette consumption (0, 1–20, >20), alcohol consumption (continuous), drink alcohol (no, yes), total protein intake (continuous), total carbohydrates intake (continuous), energy-adjusted consumption of vegetables (continuous), energy-adjusted consumption of fruits (continuous) and total energy intake (continuous). ^2^Trend test was performed using median value of FQI quintile as a continuous variable. ^3^The extreme energy intake was defined as >4,000 kcal/day or <500 kcal/day; ^4^The extreme BMI (baseline) was defined as top 1% or bottom 1% in the included population.

Consistent with the composition of a high-FQI diet, our study found that higher intakes of MUFA and PUFA were protectively associated with HNC, particularly oral and pharyngeal cancers, aligning with prior research (30). Conversely, the non-significant positive trends for SFA and TFA in our study are directionally consistent with larger cohorts that link these fats to increased cancer risk and mortality (31, 32). These epidemiological associations are biologically plausible. Unsaturated fatty acids may confer protection through several mechanisms: PUFAs can induce cancer cell death via ferroptosis (33–36), while MUFAs have been shown to trigger apoptosis in oral squamous cell carcinoma, a major HNC subtype (37). Crucially, both fat types exert potent anti-inflammatory effects by inhibiting the pro-inflammatory NF-κB pathway (10, 38). This is highly relevant, given that chronic inflammation is a key driver of HNC (39, 40). In stark contrast, TFAs and SFAs can activate this same NF-κB pathway, promoting a pro-inflammatory tumor microenvironment (41). This interplay of opposing mechanisms—the anti-inflammatory effects of unsaturated fats versus the pro-inflammatory potential of saturated and trans fats—may explain why the overall FQI’s association was attenuated for oral and pharyngeal cancers in our analysis. Taken together, this convergence of evidence suggests that improving dietary fat quality is a key modifiable factor in HNC prevention.

This study further investigated the joint association between the proportion of fat intake and the FQI in relation to HNC risk. Overall, a higher FQI was inversely associated with HNC risk, and this association appeared more pronounced among individuals with higher fat intake (≥32% of total energy). Although the protective trend of high FQI was most evident in the context of higher fat consumption, the interaction between FQI and fat intake was not statistically significant, suggesting that the inverse relationship between FQI and HNC risk was largely consistent across different levels of fat intake. Notably, a previous study based on the same cohort reported a significantly reduced risk of HNC in association with low-fat diets, highlighting the importance of limiting total fat intake (8). The current findings expand upon this evidence by demonstrating that even among individuals with relatively high fat intake, a higher FQI was still associated with a lower risk of HNC. This observation suggests that a diet rich in unsaturated fatty acids may confer anti-inflammatory benefits, potentially mitigating the deleterious effects of high total fat consumption (10, 38).

Taken together, these findings underscore a critical shift in dietary intervention strategies—from focusing solely on the quantity of fat consumed to emphasizing the quality of fat. In future public health and nutritional guidelines, improving fat quality and increasing the proportion of unsaturated fatty acids may offer more practical and effective benefits than restricting total fat intake alone.

This study possesses several methodological strengths that collectively enhance the validity and relevance of its findings. By leveraging a large-scale, well-characterized prospective cohort with extended follow-up, we were able to establish a temporally sound link between dietary fat quality and the incidence of HNC, while minimizing recall and selection bias. The use of a composite FQI, rather than isolated nutrient metrics, reflects a more holistic dietary assessment strategy that aligns with contemporary approaches in nutritional epidemiology. This allows for a more ecologically valid interpretation of dietary exposures in real-world settings. Furthermore, the comprehensive adjustment for a wide range of demographic, lifestyle, and dietary covariates—alongside multiple sensitivity analyses—supports the robustness and consistency of the observed associations.

Nonetheless, several limitations merit consideration. Dietary intake was assessed via a self-administered food frequency questionnaire, which, despite prior validation, is inherently prone to measurement error and may not capture long-term or evolving dietary habits (42). Additionally, although extensive confounder control was implemented, the possibility of residual confounding cannot be excluded, particularly for unmeasured variables such as HPV status, oral hygiene, or occupational exposures. Finally, the study population—composed predominantly of older, non-Hispanic White individuals in the United States—may limit the generalizability of our findings to more diverse populations or cultural contexts. These limitations underscore the need for future studies in other cohorts and settings to validate and expand upon our observations.

## 5 Conclusion

In conclusion, our large prospective study provides compelling evidence that improving dietary fat quality is strongly associated with a reduced risk of HNC. Crucially, this protective effect not only persists but is also more pronounced among individuals with high total fat intake. While these findings warrant confirmation in more diverse populations, they signal a pivotal shift for public health: from a singular focus on restricting fat *quantity* to a more impactful strategy of prioritizing fat *quality*. Looking ahead, an important next step is to translate these findings into clinical practice. Future studies should test whether integrating fat-quality–focused dietary counselling into routine care for high-risk individuals can reduce the incidence of HNC and related outcomes in real-world settings, thereby informing evidence-based prevention strategies.

## Data Availability

The raw data used in this article is not available because of the National Cancer Institute’s data policy. Access to the dataset should contact the National Cancer Institute by mail. Requests to access these datasets should be directed to https://cdas.cancer.gov/plco/.
